# Evasion of Apoptosis as a Cellular Stress Response in Cancer

**DOI:** 10.1155/2010/370835

**Published:** 2010-02-18

**Authors:** Simone Fulda

**Affiliations:** Ulm University, Children's Hospital, Eythstraße 24, 89075 Ulm, Germany

## Abstract

One of the hallmarks of human cancers is the intrinsic or acquired resistance to apoptosis. Evasion of apoptosis can be part of a cellular stress response to ensure the cell's survival upon exposure to stressful stimuli. Apoptosis resistance may contribute to carcinogenesis, tumor progression, and also treatment resistance, since most current anticancer therapies including chemotherapy as well as radio- and immunotherapies primarily act by activating cell death pathways including apoptosis in cancer cells. Hence, a better understanding of the molecular mechanisms regarding how cellular stress stimuli trigger antiapoptotic mechanisms and how this contributes to tumor resistance to apoptotic cell death is expected to provide the basis for a rational approach to overcome apoptosis resistance mechanisms in cancers.

## 1. Introduction

Tissue homeostasis is characterized by the balance between proliferation and cell growth on one side and cell death on the other side [1]. In response to stressful stimuli, cells usually mount a cellular stress response to ensure survival [2]. Under physiological conditions, such a stress response limits tissue damage. However, in cancer cells activation of pathways that favor cell survival instead of cell death under stressful conditions may contribute to tumorigenesis. In addition, this adaptive stress response promotes the development of acquired resistance, since current treatment approaches such as chemotherapy and irradiation trigger cellular stress pathways, and thus, initiate the activation of survival cascades and anti-apoptotic mechanisms [3]. Apoptosis or programmed cell death is the cell's intrinsic death program that regulates various physiological as well as pathological processes and that is evolutionary highly conserved [1]. Hence, further insights into the molecular mechanisms of how cellular stress signals trigger anti-apoptotic mechanisms and how this contributes to tumor resistance to apoptotic cell death are expected to provide the basis for a rational approach for the development of new molecular targeted therapies.

## 2. Signaling to Apoptotic Cell Death and Cellular Stress

There are two major apoptosis signaling pathways, that is, the death receptor (extrinsic) pathway and the mitochondrial (intrinsic) pathway [4]. Under most circumstances, activation of either pathway eventually leads to proteolytic cleavage and thus activation of caspases, a family of cysteine proteases that act as common death effector molecules [5]. Accordingly, caspases are responsible for many of the biochemical and morphological hallmarks of apoptotic cell death by cleaving a range of substrates in the cytoplasm or nucleus [5]. Ligation of death receptors of the tumor necrosis factor (TNF) receptor superfamily such as CD95 (APO-1/Fas) or TRAIL receptors by their corresponding natural ligands, that is, CD95 ligand or TRAIL, results in the recruitment of caspase-8 into a multimeric complex at the plasma membrane, the death-inducing signaling complex (DISC) [6, 7]. This in turn leads to caspase-8 activation, which can then directly cleave downstream effector caspases such as caspase-3 [7]. Alternatively, caspase-8 can promote outer mitochondrial membrane permeabilization by cleaving Bid, a BH3-only protein that translocates to mitochondria upon cleavage and causes cytochrome  c release [8]. The mitochondrial pathway is initiated by the release of apoptogenic factors such as cytochrome c, apoptosis-inducing factor (AIF) second mitochondria-derived activator of caspase (Smac)/direct IAP Binding protein with Low pI (DIABLO) or Omi/high temperature requirement protein A (HtrA2) from the mitochondrial intermembrane space into the cytosol [9]. The release of cytochrome c into the cytosol triggers activation of caspase-3 via the formation of a large cytosolic complex, which is called the apoptosome and consists of cytochrome c, Apaf-1, and caspase-9 [9]. Smac/DIABLO or Omi/HtrA2 promotes caspase activation by binding to Inhibitor of Apoptosis (IAP) proteins and thereby disrupts the interaction of IAPs with caspase-3 or -9 [9, 10]. Accidental stimulation of the apoptotic machinery can have detrimental effects on cell survival. Therefore, cancer cells react to cellular stress signals by mounting an anti-apoptotic response, which enables cancer cells to evade apoptotic cell death and ensures cell survival [11]. A wide range of stress signals has been identified, which may evoke a cell survival program in case of sublethal damage, while cell death is usually initiated if the damage is too severe, that is, starvation, hypoxia, DNA damaging drugs, irradiation, ER stress, and reactive oxygen species just to name a few [2]. 

The molecular mechanisms that initiate cell death upon cellular stress stimuli have often not exactly been identified and likely depend on the individual stimulus. For example, following exposure to genotoxic substances, damage to DNA or to other critical molecules is considered to be a common initial event which is then transmitted by the cellular stress response to the activation of cellular effector systems such as the apoptotic machinery [12]. Various stress-inducible molecules, for example, JNK, MAPK/ERK, NF-*κ*B, or ceramide have been implicated in propagating the apoptotic signal [13–15]. 

Besides caspase-dependent and caspase-independent apoptosis, additional non-apoptotic modes of cell death also exist and have gained increasing attention over the last years, including necrosis, autophagy, mitotic catastrophe, and lysosomal cell death [16, 17]. While resistance to these cell death modalities can also contribute to evasion of cell death under stress conditions, the discussion of these alternative modes of cell death is beyond the scope of this review.

## 3. Evasion of Apoptosis in Response to Cellular Stress in Cancers

**Figure 1:**
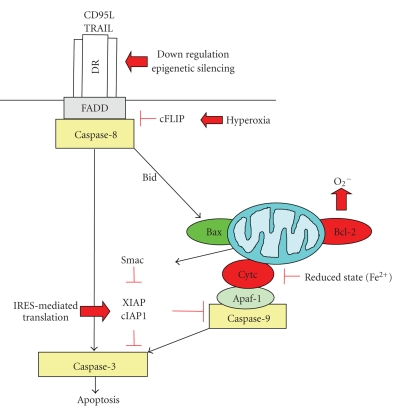
Interplay of cellular stress signals with apoptosis pathways. Signaling via the death receptor pathway can be inhibited by downregulation or epigenetic silencing of death receptors (DRs) or upregulation of cFLIP by hyperoxia. In the mitochondrial pathway, Bcl-2 favors a prooxidant milieu that promotes survival, while the reduced form of cytochrome c is inhibited in its activity to trigger caspase activation via the apoptosome. At the postmitochondrial level, translation of XIAP and cIAP1 is sustained via an IRES-dependent mechanism even under cellular stress conditions. See text for more details.

A characteristic feature of human cancers is the evasion of apoptosis in response to stress stimuli, which contributes to both tumorigenesis and treatment resistance [18]. In principle, cell death pathways can be blocked at different levels of the signaling cascade by upregulation of anti-apoptotic proteins and/or by downregulation or dysfunction of proapoptotic molecules. Examples of altered apoptosis signaling pathways that contribute to stress resistance in human cancers will be discussed in the following paragraphs (Figure 1). 

### 3.1. Evasion of the Death Receptor Pathway

Death receptors are part of the tumor necrosis factor (TNF) receptor gene superfamily, which comprises more than 20 proteins, for example, CD95 (APO − 1/Fas), TRAIL receptors, and TNF receptor 1 (TNFRI) [7, 19]. Death receptors exert many different biological functions, including the regulation of cell death and survival, differentiation, and immune regulation [7, 19]. Members of the TNF receptor family share a characteristic cytoplasmic domain called the “death domain,” which is pivotal for transducing the death signal from the cell's surface to intracellular signaling pathways [7, 19].

Signaling via death receptor can be impaired in human cancers via downregulation of receptor surface expression as part of an adaptive stress response. For example, in chemotherapy-resistant leukemia or neuroblastoma cells, downregulation of CD95 expression was identified as a mechanism of acquired drug resistance [20, 21]. For the apoptosis-inducing TRAIL receptors TRAIL-R1 and TRAIL-R2, abnormal transport from intracellular stores such as the endoplasmatic reticulum to the cell surface rendered colon carcinoma cells resistant to TRAIL-induced cell death [22]. Further, membrane expression of death receptors can be reduced by epigenetic changes such as CpG-island hypermethylation of gene promoters in response to stress signals [23, 24].

Abnormal expression of decoy receptors presents an alternative mechanism of resistance to TRAIL- or CD95-induced apoptosis. To this end, the decoy receptor 3 (DcR3), which counteracts CD95-mediated apoptosis by competitively binding CD95 ligand, was shown to be overexpressed in lung carcinoma or colon carcinoma and in glioblastoma [25, 26] and TRAIL-R3; a decoy receptor for TRAIL was reported to be expressed at high levels in gastric carcinoma [27].

In addition, anti-apoptotic proteins with a death effector domain (DED) such as cellular FLICE-Inhibitory Protein (cFLIP) and phosphoprotein enriched in diabetes/ phosphoprotein enriched in astrocytes-15 kDa (PED/PEA-15) can be aberrantly expressed upon cellular stress [28, 29]. For example, high oxygen tension (hyperoxia) has been reported to lead to upregulation of cFLIP, which inhibited apoptosis during hyperoxia by suppressing both extrinsic and intrinsic apoptotic pathways, the latter via inhibition of Bax [30]. Because of their sequence homology to caspase-8, both cFLIP and PED can be recruited into the death-inducing signaling complex (DISC) upon receptor ligation instead of procaspase-8, thereby preventing caspase-8 activation [28, 29].

Moreover, the expression of caspase-8 or its function is impaired by genetic or epigenetic mechanisms in various cancers. For example, caspase-8 mutations were identified in some tumors, that is, in colorectal and head and neck carcinomas, although the overall frequency is low [31, 32]. In addition, homo- or heterozygous genomic deletions were detected in neuroblastoma [33]. Alternative splicing of intron 8 of the caspase-8 gene resulting in the generation of caspase-8L, a catalytically inactive splice variant presents another mechanism of caspase-8 inactivation [34, 35]. Epigenetic silencing secondary to hypermethylation of regulatory sequences of the caspase-8 gene occurs in various tumors, for example, neuroblastoma, malignant brain tumors, Ewing tumor, retinoblastoma, rhabdomyosarcoma, or small lung cell carcinoma [33, 36–39]. Furthermore, phosphorylation of caspase-8 on tyrosine 308 by, for example, Src has been shown to interfere with its proapoptotic activity [40].

### 3.2. Evasion of the Mitochondrial Pathway

#### 3.2.1. Bcl-2 Family Proteins

The Bcl-2 family of proteins consists of both anti-apoptotic proteins, for example, Bcl-2, Bcl-*X*_*L*_, and Mcl-1, as well as proapoptotic molecules such as Bax, Bak, and BH3 domain only molecules [8]. There are currently two models to explain the activation of Bax and Bak by BH3-only proteins. The direct activation model holds that BH3-only proteins, which act as direct activators such as Bim and the cleaved form of Bid (tBid), bind directly to Bax and Bak to trigger their activation, while BH3-only proteins that act as sensitizers, for example, Bad, bind to the prosurvival Bcl-2 proteins [41]. According to the indirect activation model, BH3-only proteins activate Bax and Bak in an indirect fashion by engaging the multiple anti-apoptotic Bcl-2 proteins that inhibit Bax and Bak, thereby releasing their inhibition on Bax and Bak [42, 43]. Regardless of the exact mode of Bax and Bak activation, the ratio of anti-apoptotic versus proapoptotic Bcl-2 proteins rather than the expression levels of one particular molecule of the Bcl-2 family regulates apoptosis sensitivity. 

An increase in the ratio of anti- to proapoptotic Bcl-2 proteins has been detected in various cancers and has been correlated to tumor cell survival and apoptosis resistance. More recently, Bcl-2 has also been implicated in the regulation of the intracellular redox status [44]. Bcl-2 localizes to mitochondrial membranes as well as the endoplasmatic reticulum and the nuclear envelope, which are all sites of ROS production [45]. While Bcl-2 has initially been described as an anti-oxidant because of its inhibitory effect on H_2_O_2_-induced lipid peroxidation [46], there is also evidence that Bcl-2 may promote a prooxidant intracellular milieu. Accordingly, ectopic expression of Bcl-2 resulted in an elevated constitutive level of superoxide anion and intracellular pH in leukemia cells [47]. Conversely, reduction of intracellular superoxide sensitized Bcl-2-overexpressing tumor cells to apoptotic stimuli independent of the mitochondria [47]. These findings provide a link between oncogene-mediated alterations in the intracellular redox status and cell survival. 

#### 3.2.2. Cytochrome c

Besides Bcl-2, also cytochrome c has been implicated in the redox regulation of apoptosis. Once cytochrome c is released from mitochondria into the cytosol, it triggers formation of the cytochrome c/Apaf-1/Caspase-9-containing apoptosome, which in turn lead to activation of caspase-9 and downstream effector caspases [48]. There is recent evidence that also the redox state of cytochrome c is involved in the regulation of apoptosis. To this end, the oxidized form of cytochrome c (Fe(3+)) has been reported to induce caspase activation via the apoptosome, while the reduced form of cytochrome c (Fe(2+)) is unable to do so [49–51]. Several mechanisms have been discussed to be responsible for this redox-mediated regulation of cytochrome c activity, including different affinities of the oxidized versus the reduced form of cytochrome c for binding to Apaf-1, different abilities of these cytochrome c forms to activate Apaf-1, or, alternatively, different affinities for other factors not belonging to the apoptosome. Regardless of the exact mechanisms, this regulation of the redox state of cytochrome c opens the possibility of controlling the effector phase of apoptosis at a postmitochondrial level. 

Besides these genetic alterations in Bcl-2 family proteins, impairment of mitochondrial apoptosis may also occur at the postmitochondrial level. For example, expression level or activity of Apaf-1 may be reduced due to promoter hypermethylation or loss of heterozygosity at chromosome 12q22-23, which in turn leads to impaired assembly of a functional apoptosome [52–56]. 

### 3.3. Evasion of Apoptosis via Aberrant Expression of “Inhibitor of Apoptosis” (IAP) Proteins

Moreover, tumor resistance to apoptosis may be caused by aberrant expression or function of “Inhibitor of Apoptosis” (IAP) proteins. IAP proteins are a family of endogenous caspase inhibitors with eight human members, that is, XIAP, cIAP1, cIAP2, survivin, livin (ML-IAP), NAIP, Bruce (apollon), and ILP-2 [10, 57]. All IAP proteins have at least one baculovirus IAP repeat (BIR) domain that is required for classification as IAP family protein. This domain is also the region of the protein that mediates the interaction with caspases [58]. Among the IAP family proteins, XIAP exhibits the strongest anti-apoptotic properties and inhibits apoptosis signaling by binding to active caspase-3 and -7 and by preventing caspase-9 activation [59]. 

The expression and function of IAP proteins are tightly regulated by several mechanisms, among them is translational regulation [60]. To this end, it is particularly interesting to note that XIAP and cIAP1 belong to the proteins, which are translated via an internal ribosome entry site (IRES). This unique property enables protein translation of these IAP proteins even under cellular stress conditions when protein synthesis is usually shut down, for example, because of caspase-dependent breakdown of eukaryotic translation initiation factors coupled with activation of the double-stranded RNA-activated protein kinase PKR [61].

Typically, mRNA molecules are translated via a cap-dependent translation mechanism [62]. However, the mRNAs encoding XIAP or cIAP1 protein contain very long 5′ untranslated regions (UTRs), which are not amenable to a ribosome-scanning translation initiation mechanism and thus, require a cap-independent translation initiation mechanism, that is, IRES-mediated translation [60]. IRES-mediated translation allows for the continued translation of XIAP and cIAP1 even under conditions where cap-dependent translation is inhibited such as cellular stress [60]. In addition, IRES-mediated translational regulation of XIAP and cIAP1 expression enables a rapid response to transient cellular stress conditions in order to delay cell death and ensure survival. Of note, cellular stress signals, including low-dose irradiation, anoxia, serum starvation and chemotherapeutic drugs, have been reported to stimulate the IRES activity of XIAP or cIAP1 [63–66]. This is in line with the concept that such stress signals promote cell survival under stress conditions, at least in part, via IRES-mediated upregulation of anti-apoptotic proteins.

## 4. Conclusions

Evasion of apoptosis is one of the hallmarks of human cancers that promote tumor formation and progression as well as treatment resistance. Cellular stress signals can contribute to evasion of apoptosis by activating anti-apoptotic and cell survival programs that ultimately block cell death. This interference with proper apoptosis signaling under stress conditions can occur at different points of the apoptosis signaling network, for example, within the death receptor or the mitochondrial pathway or at the postmitochondrial level. Whether or not cellular stress eventually engages cell survival or cell death programs also depends on the type and strength of the stress stimulus as well as the cell type. A better understanding of the molecular mechanisms of this interplay between the cellular stress response and anti-apoptotic programs is expected to yield novel molecular targets for therapeutic interventions. The aim is to prevent protective responses in order to maximize the antitumor activity of anticancer treatment approaches. This strategy will hopefully lead to more effective treatment options for cancer patients.
